# The tolerability of single low dose primaquine in glucose-6-phosphate deficient and normal falciparum-infected Cambodians

**DOI:** 10.1186/s12879-019-3862-1

**Published:** 2019-03-12

**Authors:** Lek Dysoley, Saorin Kim, Sergio Lopes, Nimol Khim, Steven Bjorges, Samphornarann Top, Chea Huch, Huy Rekol, Nelli Westercamp, Mark M. Fukuda, Jimee Hwang, Arantxa Roca-Feltrer, Mavuto Mukaka, Didier Menard, Walter R. Taylor

**Affiliations:** 1 National Center for National Centre for Parasitology, Entomology and Malaria Control, Phnom Penh, Cambodia; 2 School of Public Health, National Institute of Public Health, Phnom Penh, Cambodia; 3 Institut Pasteur du Cambodge, Phnom Penh, Cambodia; 4 Malaria Consortium, Phnom Penh, Cambodia; 5 WHO Cambodia country office, Pasteur Street, Phnom Penh, Cambodia; 6 Malaria Branch, Centers for Disease Control and Prevention, 1600 Clifton Rd, Atlanta, GA 30333 USA; 7 U.S. President’s Malaria Initiative, Malaria Branch, Division Parasitic Diseases and Malaria, Centers for Disease Control and Prevention, Bangkok, Thailand; 8 U.S. President’s Malaria Initiative, Malaria Branch, Division Parasitic Diseases and Malaria, Centers for Disease Control and Prevention, Atlanta, GA USA; 9 Mahidol Oxford Tropical Medicine Research unit (MORU), 420/60 Rajvithi Road, Bangkok, 10400 Thailand; 10 Centre for Tropical Medicine, Nuffield Department of Medicine, University of Oxford, Oxford, UK; 11 Biology of Host-Parasite Interactions Unit, Malaria Genetics and Resistance Group, Institut Pasteur - INSERM U1201 - CNRS ERL9195, Paris, France

**Keywords:** Malaria, Transmission blocking, Primaquine, G6PD deficiency, Cambodia

## Abstract

**Background:**

The WHO recommends single low-dose primaquine (SLDPQ, 0.25 mg/kg body weight) in falciparum-infected patients to block malaria transmission and contribute to eliminating multidrug resistant *Plasmodium falciparum* from the Greater Mekong Sub region (GMS). However, the anxiety regarding PQ-induced acute haemolytic anaemia in glucose-6-phosphate dehydrogenase deficiency (G6PDd) has hindered its use. Therefore, we assessed the tolerability of SLDPQ in Cambodia to inform national policy.

**Methods:**

This open randomised trial of dihydroartemisinin-piperaquine (DHAPP) + SLDPQ vs. DHAPP alone recruited Cambodians aged ≥1 year with acute uncomplicated *P. falciparum*. Randomisation was 4:1 DHAPP+SLDPQ: DHAPP for G6PDd patients and 1:1 for G6PDn patients, according to the results of the qualitative fluorescent spot test. Definitive G6PD status was determined by genotyping. Day (D) 7 haemoglobin (Hb) concentration was the primary outcome measure.

**Results:**

One hundred nine patients (88 males, 21 females), aged 4–76 years (median 23) were enrolled; 12 were G6PDd Viangchan (9 hemizygous males, 3 heterozygous females). Mean nadir Hb occurred on D7 [11.6 (range 6.4 ─ 15.6) g/dL] and was significantly lower (*p* = 0.040) in G6PDd (*n* = 9) vs. G6PDn (*n* = 46) DHAPP+SLDPQ recipients: 10.9 vs. 12.05 g/dL, Δ = -1.15 (95% CI: -2.24 ─ -0.05) g/dL. Three G6PDn patients had D7 Hb concentrations < 8 g/dL; D7-D0 Hbs were 6.4 ─ 6.9, 7.4 ─ 7.4, and 7.5 ─ 8.2 g/dL.

For all patients, mean (range) D7-D0 Hb decline was -1.45 (-4.8 ─ 2.4) g/dL, associated significantly with higher D0 Hb, higher D0 parasitaemia, and receiving DHAPP; G6PDd was not a factor. No patient required a blood transfusion.

**Conclusions:**

DHAPP+SLDPQ was associated with modest Hb declines in G6PD Viangchan, a moderately severe variant. Our data augment growing evidence that SLDPQ in SE Asia is well tolerated and appears safe in G6PDd patients. Cambodia is now deploying SLDPQ and this should encourage other GMS countries to follow suit.

**Trial registration:**

The clinicaltrials.gov reference number is NCT02434952.

**Electronic supplementary material:**

The online version of this article (10.1186/s12879-019-3862-1) contains supplementary material, which is available to authorized users.

## Background

Despite the 2012 WHO recommendation to add single low dose primaquine (SLDPQ, 0.25 mg/kg body weight) to artemisinin-based combination therapies (ACTs) for blocking the transmission of *Plasmodium falciparum* in sick patients, few countries have introduced SDLPQ in their treatment guidelines. Reluctance has centred on the risk of precipitating dose-dependent, acute haemolytic anaemia (AHA) in patients with glucose-6-phosphate dehydrogenase deficiency (G6PDd), the logistical difficulty in identifying G6PDd patients at the point of care, and the lack of suitable, paediatric PQ tablet strengths and formulations [1].

PQ’s oxidative metabolites induce oxidant stress leading to damage of red blood cell membranes and acute extra- and intravascular haemolysis [2–4]. AHA is more severe in the more severe G6PDd variants like Viangchan, Mahidol, Coimbra, Canton, Kaiping that are found in SE Asia [5–7], and the Mediterranean variant, found in the Middle East [8]. Moreover, because G6PD hemizygous males and homozygous females have the lowest G6PD enzyme activities, they are most prone to AHA [9]. The risk of clinically significant AHA, which may require blood transfusion, is in the first week when most malaria patients and healthy subjects given PQ reach their nadir haemoglobin (Hb) concentration [10–14].

The safety evidence base of SLDPQ in G6PDd individuals is growing, including an ongoing trial in falciparum-infected African children (ISRCTN11594437). The mild A- (202, 376) G6PD variant predominates in Africa [8, 15] and this limits the generalisability of Hb dynamic data to SE Asia. Nevertheless, several studies demonstrate a common theme. When different PQ doses (0.25, 0.4 or 0.75 mg/kg) are given with ACTs, there is a greater initial fall in mean Hb concentrations in both symptomatic and asymptomatic, G6PDd malaria infected patients and G6PDd healthy individuals compared to G6PD normal (n) patients and individuals [16–22].

SLDPQ data from SE Asia are limited. One study demonstrated reassuringly modest Hb declines in healthy Burmese individuals of all ages with ‘good’ mean baseline Hb concentrations [10.8 (< 5y), 11.6 (6-15y), 13.4 (adult males) and 11.7 (adult females) g/dL] who underwent mass drug administration with SLDPQ. Mean (upper 95% confidence interval) fractional falls of 5 (9) % and 1.7 (4) % were seen in the G6PDd and G6PDn individuals, respectively [23].

In falciparum-infected, Cambodian adults, 45 mg of PQ (0.75 mg/kg) was given after dihydroartemisinin- piperaquine (DHAPP) at 72 h [Day (D) 3] and showed a possible trend (*p* = 0.14) of a greater fractional D7-D0 Hb decline in six G6PDd DHAPP+PQ vs. two G6PDd DHAPP recipients; maximal declines were 29.8 and 4.4%, respectively [24]. Weekly PQ (0.75 mg/kg/w x 8w) was well tolerated in 18 G6PDd and 57 G6PDn Cambodians with vivax malaria [12]. Median nadir Hb concentrations occurred on D2 in both groups, rising on D3 (G6PDn) and D7 (G6PDd); median (range) G6PDd vs. G6PDn D7-D0 Hb declines were − 2.2 (− 4.9 ─ 0.8) vs. -0.5 (− 2.2 ─ 2.8) g/dL (*p* = 0.0002). One G6PDd male was transfused for symptomatic anaemia of 7.2 g/dL (D0 Hb 10 g/dL). Methaemoglobin (metHb) increases, another dose- and oxidant-related toxicity of PQ, were modest, reaching a maximum of 4.9%. Smithuis et al gave single high dose PQ, 0.75 mg/kg, to 397 malaria infected patients of all ages who were treated with four ACTs in Myanmar where the moderately severe Mahidol variant has a prevalence of ~10% [6]; PQ was well tolerated but G6PD status not determined and Hb measured only on D0 and D63 [25].

The study by Kheng et al [12] encouraged the Cambodian National Malaria Control program (CNM) to conduct a trial of SLDPQ tolerability in uncomplicated falciparum patients to inform drug policy.

## Methods

### Study site

The study took place from February 2015 to August 2016 in Banlung, Ratanakiri province, NE Cambodia, an area of mixed falciparum vivax transmission. From 2010 to 14, *P. falciparum* (*Pf*) cases fell from ~69 ─ ~16/1000 population and *P. vivax* (*Pv*) from ~16 ─ ~11/1000 [26].

G6PD Viangchan is the predominant variant in Cambodia, occurring at rates of ~15 and 9% in western and eastern Cambodia, respectively. Measured enzyme activities in hemizygous males and homozygous females are ~0.5 ─ < 3.6 U/g Hb, equivalent to ~4.1 ─ < 30% of 12 U/g Hb, the G6PDn population median [7, 27]. Haemoglobin E is common, ~40% prevalence, and occurs equally in *Pf*- and *Pv*-infected patients [27].

### Study design & participants

This was a randomised open, parallel safety trial. The inclusion criteria were patients aged ≥1 year (y) and weighing ≥7 kg with acute (≤ 48 h), symptomatic (≥ 38 °C axilla/≥ 37.5°C aural/history of fever), uncomplicated falciparum malaria (≥ 1 asexual form/500 white blood cells) who or whose legal guardian gave signed informed consent and children aged age 12 to < 18 y signed an assent form.

Excluding criteria were: (i) clinical signs of severe malaria/danger signs, (ii) Hb < 5 g/dL or < 6 g/dL + significant anaemia-related symptoms, (iii) pregnant/breast feeding, (iv) unable/unwilling to take a pregnancy test (women of child-bearing age), (v) intentional pregnancy in coming 3 months, (vi) allergic to PQ/DHAPP, (vii) on known haemolysing drugs in G6PDd, (viii) a significant concurrent illness/infection +/− treatment e.g. HIV, TB treatment, steroids, and (ix) on drugs influencing PQ/DHAPP pharmacokinetics e.g., antiretrovirals, cimetidine, antiepileptics.

The Cambodian National Ethics Committee approved the study, which also underwent human subjects review at the U.S. Centers for Disease Control and Prevention and was approved as non-engagement in human subjects research. The trial registration reference is NCT02434952.

### Conduct of clinical trial

#### Interventions

All enrolled patients were randomised to receive DHAPP (Eurartesim®, Sigma Tau, Pomezia, Italy), dosed by weight following the manufacturer’s instructions; in July, the dose was changed for children < 25 kg, following new WHO guidelines, so that they received a DHA target dose of 2.5 mg/kg. This dosing change affected 10 children: 5 received the Sigma Tau dose and 5 the WHO dose. Only the largest DHAPP tablet strength (DHA 40 mg, PP 320 mg) was available in Cambodia, necessitating the use of tablet fractions.

Primaquine tablets, containing 15 mg of PQ base (Government Pharmaceutical Organisation, Bangkok, Thailand), were dosed by weight. The target dose was 0.25 mg base/kg and tablet fractions were used as needed. For small children, PQ tablets were dissolved in 5 mL of water (3 mg/mL) and dosed accordingly. Patients were observed for early (≤ 1 h) vomiting and a full or half dose of both drugs were readministered within 30 and 60 min, respectively.

#### Evaluations

Patients were admitted to the research clinic for the first 3 days for supervised treatment and investigations. Baseline procedures included a history of illness, recent drug intake, family history, symptom questionnaire, physical examination, and laboratory evaluations. Follow up was limited to 28 days because the focus of this safety study was the initial fall in and early recovery of Hb.

On study Days (D) 0, 1, 2, 3, 7, 14, and 28, the following were done: (i) symptom checklist, (ii) vital signs, metHb [transcutaneous Masimo RAD 57 oximeter (Masimo, Irvine, CA)], (iii) Hb (HemoCue® Angelholm, Sweden), (iv) Giemsa-stained malaria blood films, read under × 1000 magnification; a negative thick film was declared after reading 8000 white blood cells, (v) thin blood films for red cell morphology and reticulocyte count, and (vi) dried blood spots for: (a) malaria genotype (*msp1, msp2, glurp* genes) to differentiate recurrent parasitaemia, (b) PQ concentrations (D0, + 1 h, + 2 h, + 8 h, + 12 h & + 24 h), and (c) piperaquine concentrations (D0, D7, D of recurrent parasitaemia).

Other samples taken were for: (i) in vitro parasite drug sensitivity (D0), (ii) full blood count: D0, 7, 14, 28 [ABX Pentra XL 80 (Horiba Medical, Irvine, CA], (iii) Hb (WHO Hb colour chart: D0, 7, 14, 28), and (iv) biochemistry (D0 & 28), using a Pentra C400 (Horiba Medical, Irvine, CA).

G6PD status in the field was diagnosed initially using the fluorescent spot test (FST) to allow for the allocation of SLDPQ (see Randomisation below). The qualitative, Carestart® (AccessBio, Somerset, NJ), rapid diagnostic test (RDT) was assessed in parallel. Both tests detect reliably enzyme activities < 30% of a normal male population and have a high degree of concordance [7, 28].

G6PD enzyme activity was measured by the Trinity Biotech quantitative G6PD assay™ (Trinity Biotech, Jamestown, NY), adapted on an Integra 400 analyser™ (Roche Diagnostic, Basel, Switzerland)]. Fresh samples were sent on ice from the site to Phnom Penh where the Hb type was also determined by Hb electrophoresis (MINICAP system™, Sebia, Evry, France).

### G6PD genotyping

Common SE Asian G6PD gene mutations were sought in selected patients with G6PD enzyme activities ≤12.3 U/g Hb (i.e. just above the normal population median of 12 U/g Hb [7]): (i) in exon 6 for the Mahidol (487G > A), Mediterranean (563C > T) and Coimbra (592C > T) variants, (ii) in exon 9 for the Viangchan (871G > A) and Chinese-5 (1024C > T) variants, (iii) in exon 11 for the Union (1360C > T) variant, and (iv) in exon 12 for the Canton (1376G > T) variant. All other patients with G6PD activity > 12.3 U/g Hb were classed as G6PD wild type (normal).

### Outcomes

The primary outcome was the D7 Hb concentration. The main secondary outcomes included: (i) D7-D0 absolute and fractional falls, (ii) modelled Hb changes over time, (iii) total malaria attributable fall (MAFt): D28Hb-nadirHb, (iv) Hb recovery (D28 Hb > D0 Hb concentration), (v) G6PD geno- and phenotype, thalassemia type, [29]), (vi) D28 cure rate, (vii) gametocyte carriage, and (viii) PQ, carboxyPQ, and PP concentrations.

### Tolerability, adverse events (AEs) and SAEs

These were assessed by an open question, symptom checklist, and a visual analogue scale graded 1 (“I/my child feel/s very bad”) to 5 (“I/my child feel/s very well”). We recorded the following AEs and SAEs: (i) early (≤ 1 h) or late (> 1 h) abdominal pain, nausea or vomiting, (ii) Hb fractional fall ≥25% vs. D0, (iii) passing dark urine graded ≥5 (Hillmen colour chart [29]), (iv) itching, (v) rash, (vi) AEs of concern (physician judgement), and (vii) any drug-related SAEs. AEs were graded using the 2004 Division of AIDS toxicity table for adults and children (https://rsc.tech-res.com/docs/default-source/safety/table_for_grading_severity_of_adult_pediatric_adverse_events.pdf).

### Randomisation & blinding

Separate computer-generated randomisation lists were used for the G6PDd and G6PDn groups. G6PDd patients were randomised 4:1 to receive DHAPP+SLDPQ and DHAPP (i.e. 4 DHAPP+SLDPQ for every one DHAPP); the G6PDn group was randomised on a 1:1 basis. The randomisation lists showed the treatment allocation (i.e. DHAPP+SLDPQ or DHAPP) and were openly accessible to the senior research team member responsible for drug allocation.

### Sample size calculation

The mean [standard deviation (SD)] D7 Hb in ~6800 ACT-treated, falciparum-infected patients of all ages from SE Asia, was 11.27 (1.74) g/dL (WRJT, unpublished). For a two-sided alpha of 0.05, power of 80%, SD of 1.74 g/dL, and assuming mean D7 Hb Δ of − 1 g/dL in the G6PDd DHAPP+SLDPQ arm vs. G6PDn DHAPP+SLDPQ arm, the sample size was 48/arm, rounded up to 50. We also decided to recruit 50 G6PDd and 50 G6PDn patients to receive DHAPP (‘control’ group).

### Data management and statistical methods

Data collected on paper case record forms were double entered into Epi Data v3.1, cleaned, then analysed in Stata v14 (Stata Corporation, College Station, TX). All patients who received ≥1 dose of DHAPP +/− SLDPQ were analysed.

ABX Pentra XL 80 measured Hb concentrations were considered the ‘gold’ standard and used for analyses involving D0, D7 and D7-D0 declines. Because HemoCue Hb measurements were more frequent, we used these results for: (i) defining the Hb nadir day, (ii) modelling Hb dynamics, (iii) MAFt, and (iv) Hb recovery, cognizant that HemoCue Hb concentrations were generally higher than those of the ABX Pentra XL 80.

Proportional data were compared using chi-squared or Fisher’s exact test, as appropriate. Student’s ‘t’ and non-parametric tests were used to assess mean and distribution differences in normally or skewed distributed continuous data, respectively. Multiple linear regression determined factors (e.g. age, sex, D0 Hb, D0 reticulocyte count, D0 parasitaemia, G6PD status, thalassaemia (grouping homo & heterozygous HbE and α and β thalassaemia) for the D7-D0 Hb fall and MAFt (backward-stepwise approach). A linear mixed effects regression model determined factors associated with changes over time in the mean reticulocyte counts, and mean Hb concentrations; analysis of covariance was adopted to adjust for the D0 Hb concentration by including it as a model covariate. Factors influencing Hb recovery were determined by logistic regression.

## Results

### Patient disposition and baseline characteristics

**Fig. 1:**
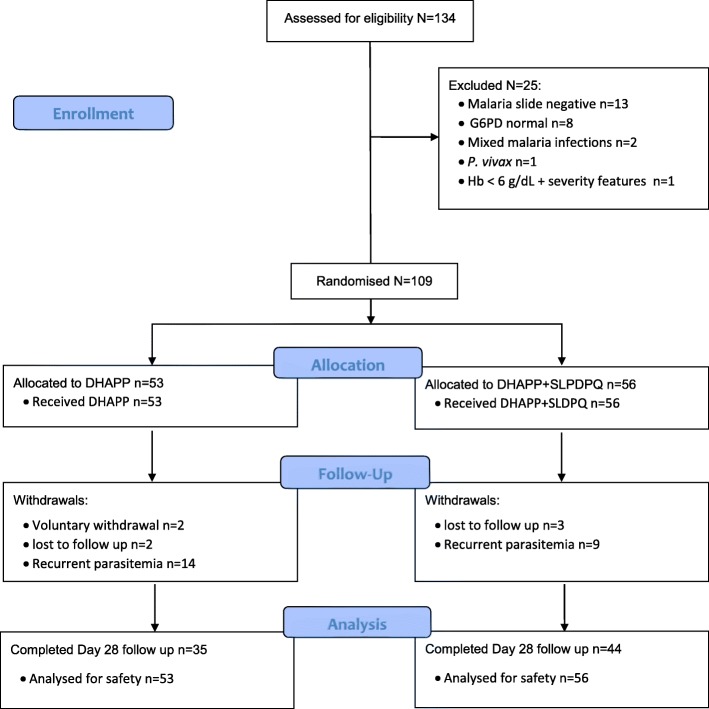
Trial profile

Of 134 patients volunteering for the trial, 109 were recruited (Fig. 1); the main disqualifying reasons were a negative malaria slide and being G6PDn after that sample size had been reached. Most patients were young male farmers in their 20s (median 23 y); the other patients were either preschool children or children at school. The youngest patient was 4 years old and pre-adolescent children (≤ 12 y) accounted for ~20% of the total. Baseline characteristics between the DHAPP and DHAPP+SLDPQ arms were essentially the same (Table 1) aside from significantly different distributions of the platelet counts and LDH concentrations (probable chance findings).

**Table 1:** Baseline characteristics by treatment arm

Parameter	DHAPP arm *N* = 53	DHAPP + SLDPQ arm *N* = 56	*P* value
Age in years	20 (14–31) [6–76]	24.5 (17.5–32) [4–65]	0.25
Age ≤ 12 y	12 (22.6)	9 (16.1)	0.38
Sex M:F	44:9	44:12	0.56
Occupation			
Farmer	40 (75.5)	46 (83.6)	0.29
Symptoms	*n* = 53	*n* = 55	
VAS score^a^	2 (2–2) [2, 3]	2 (2–2) [1–3]	0.45
Fever	52 (98.1)	53 (96.4)	0.58
Chills	51 (96.2)	53 (96.4)	0.97
Headache	49 (92.5)	54 (98.2)	0.16
Anorexia	1 (1.9)	0 (0)	0.31
Nausea	49 (92.5)	41 (74.6)	0.01
Vomiting	51 (96.2)	53 (96.4)	0.97
Abdominal pain	4 (7.6)	4 (7.3)	0.96
Palpitations	3 (5.7)	9 (16.4)	0.08
Cough	3 (5.7)	3 (5.5)	0.96
Muscle aches	4 (7.6)	7 (12.7)	0.37
Passing normal colour urine	53 (100)	55 (100)	–
Physical signs			
Temperature ^0^C	38 (38–38.5) [38–41]	38 (38–38.5) [38–40]	0.75
Weight kg	48 (36–55) [17–73]	50.5 (40–55.5) [10–74]	0.32
Respiratory rate / minute	30 (26–30) [20–34]	30 (28–30) [20–32]	0.58
Normal colour of lips	53 (100)	55 (100)	–
Pale conjunctivae/palms/tongue	0 (0)	0 (0)	–
Palpable liver	0 (0)	0 (0)	–
Palpable spleen	0 (0)	0 (0)	–
G6PD data			
G6PD activity median U/g Hb	10.45 (7.9–12.9) [0.4–17.9]	10.15 (6.7–11.7)[0.4–25]	0.23
G6PD deficient by FST^b^	3 (5.7)	6 (10.9)	0.49
G6PD deficient by RDT^c^	3 (5.7)	6 (10.9)	0.49
G6PD Viangchan	3	9 (42.8)	0.69
Haemoglobin electrophoresis	*n* = 52	*n* = 54	
Normal haemoglobin	23 (44.2)	28 (51.8)	0.34
Haemoglobin E	22 (42.3)	19 (35.2)	
Mixed Hb E alpha thalassemia	3 (5.8)	1 (1.8)	
Mixed Hb E beta thalassemia	0	3 (5.6)	
Alpha thalassemia	3 (5.8)	3 (5.6)	
Heterozygous foetal haemoglobin	1 (1.9)	0	
Full blood count			
Haemoglobin g/dL	12.63 (2.29) [6.9–18.2]	13.19 (2.1) [8.7–18]	0.19
Reticulocyte count %	1.5 (1–2)[0.4–4.2]	1.65 (1.05–2.6) [0.3–9.2]	0.13
Methaemoglobin %	1.5 (1.3–1.8) [0.7–1.9]	1.3 (1.2–1.7) [0.6–2]	0.90
Total white cell count × 10^3^/μL	5.6 (4.5–7.0) [1.5–13]	5.45 (4.45–6.3) [1.6–10.3]	0.50
***Platelet count ×10***^***3***^***/***μL	***89 (49–122) [12–336)***	***101.5 (76–156.5) [10–328]***	***0.04***
Biochemical parameters			
Total bilirubin μmol/L	15.65 (8.6–19.4) [3.7–56.1]	13.35 (10.1–22.1) [3.7–90.5]	0.87
Conjugated bilirubin μmol/L	6.35 (4.1–9.2) [1.3–16.7]	5.85 (4.45–9.25) [2.3–20.4]	0.70
Free bilirubin μmol/L	8.2 (5.2–10.9) [2.2–49.0)	7.4 (5.75–12.5) [1.4–70.1]	0.97
AST IU/L	26 (22–38) [12–133]	28 (22–38) [15–84]	0.99
ALT IU/L	21 (16–28) [6–125]	23.5 (15.5–35.5) [4–140]	0.42
***LDH IU/L***	***250 (222–347) [137–772]***	***225.5 (200–285) [59–657]***	***0.04***
Haptoglobin g/L	0.69 (0.1–1.43) [0.03–2.24]	0.59 (0.1–1.53) [0.01–2.64]	0.60
Creatinine μmol/L	63 (49–74) [29–112]	63.5 (44.5–72) [18–120]	0.53
Parasite data			
Falciparum parasitaemia N/μL	19,800 (5800-68,119) [1–350,000]	18,600 (3923–36,093 [40–800,200]	0.40
Urine			
Hillmen colour scale	3 (3–3) [1–4]	3 (3–3) [1–4]	0.57

^a^ VAS – visual analogue scale from 1 to 5

Continuous data are median (interquartile range) [full range], except the haemoglobin concentration [mean (standard deviation)

Categorical data are n (%)

^b^ Fluorescent spot test

^c^ Rapid diagnostic test

Continuous data are median (interquartile range) [full range], except the haemoglobin concentration: mean (standard deviation) [range]

Categorical data are n (%)

Entries in bold are statistically significant

Following treatment, 5 patients were lost to follow up, 2 withdrew early, and 23 had recurrent *Pf* parasitaemias between D12─28, classed as late treatment failures (LTF). LTF rates were not significantly different (*p* = 0.18) between DHAPP+SLDPQ: 9/56 (16.1%) vs. DHAPP 14/53 (26.4%) recipients.

Genotyping confirmed 12 G6PD Viangchan patients: 9 hemizygous males and 3 heterozygous females. Eight males and one female were diagnosed correctly by the FST but one male (enzyme activity 3.1 U /g Hb) and two females (7.9 & 8.4 U / g Hb) were diagnosed as G6PDn. Three patients (1 male, 2 females) who were all FST and RDT G6PDn did not have G6PD activity measurements; the male was classed as G6PDn and the two females were genotyped as G6PD wild type. Additional file 1 lists the G6PD genotype and FST data.

### Primary outcome & D7-D0 changes

For all patients, the nadir Hb occurred on D7: mean (range) 11.6 (6.4 ─ 15.6) g/dL. In the DHAPP+SLDPQ treated patients, the mean Hb was significantly (*p* = 0.040) lower in the G6PDd patients 10.9 (8.5 ─ 13.2) vs. G6PDn 12.05 (8.7 ─ 14.5) g/dL. Within the G6PDn group, D7 mean Hbs were significantly lower (*p* = 0.01) in the DHAPP vs. DHAPP+SLDPQ recipients: 11.19 vs. 12.05 g/dL, Δ = -0.86 (-1.56 ─ -0.15).

**Fig. 2:**
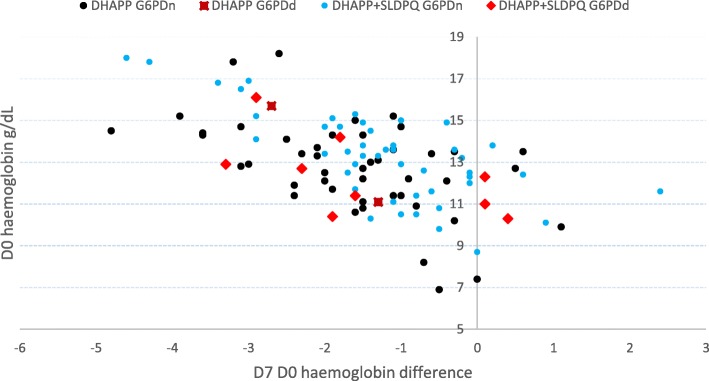
Scatterplot of baseline hemoglobin concentrations and D7─D0 declines in the patients treated with dihydroartemisinin-piperaquine +/− single low dose primaquine, by G6PD status (normal or deficient)

The absolute D7-D0 Hb declines were consistent between the G6PDd and G6PDn patients and inversely related to D0 Hb (Fig. 2): median -1.5 (mean 1.45), interquartile range (IQR) -2.1 – -0.6, and range -4.8 – 2.4 g/dL. In the DHAPP+SLDPQ arm, the mean (range) Hb declines were not significantly (*p* = 0.63) different in the G6PDd vs. G6PDn arms: -1.46 (-3.3 ─ 0.4) g/dL vs. -1.24 (-4.6 ─ 2.4) g/dL, for fractional falls of -11.2 (-25.6 ─ 3.9) % vs. -8.6 (-25.6 ─ 20.7) %.

The multiple linear regression model demonstrated an inverse relationship between the D7-D0 decline and (i) D0 Hb, and (ii) D0 parasite count (Table 2), but DHAPP+SLDPQ, relative to the DHAPP, was associated a positive change in the D7-D0 Hb, a mean increase of 0.52 (0.14 – 0.94), *p* = 0.009. These three significant factors explain 42.4% of the variation in the D7-D0 Hb decline (adjusted R^2^ = 0.4247).

**Table 2:** Independent factors associated with several markers of haemoglobin dynamics and recovery in all patients

Parameter	Coefficient	95% confidence interval	*P* value
Day 7 Day 0 decline in haemoglobin concentration			
D0 haemoglobin concentration	−0.34	−0.45– −0.24	< 0.001
D0 parasite count	−2.69 × 10^−7^	−4.81 × 10^−6^ – −5.60 × 10^− 7^	0.014
On DHAPP+ SLDPQ	0.48	0.08–0.88	0.019
Haemoglobin dynamics over 28 days			
DHAPP+SLDPQ	0.69	0.11 ─ 1.27	0.019
Age	0.034	0.014–0.055	0.001
Male	0.91	0.19–1.62	0.013
Reticulocyte dynamics	−0.12	− 0.17 – − 0.06	0.000
Total malaria attributable fall in haemoglobin			
Treatment failure	−1.28	−2.27 – −0.029	0.012
D7 haemoglobin concentration	−0.53	−0.74 – − 0.32	< 0.001
Male	0.99	0.004–1.99	0.049
Haemoglobin recovery^a^			
Male	7.2	1.3–40.0	0.025
Age	1.04	1.001–1.08	0.045
D0 haemoglobin concentration	0.51	0.34–0.76	0.001
Reticulocyte dynamics			
D0 haemoglobin concentration	−0.320	− 0.420 ─ -0.219	< 0.001

^*a*^By logistic regression, the odds ratio is reported with 95% confidence interval

### Haemoglobin dynamics

**Fig. 3:**
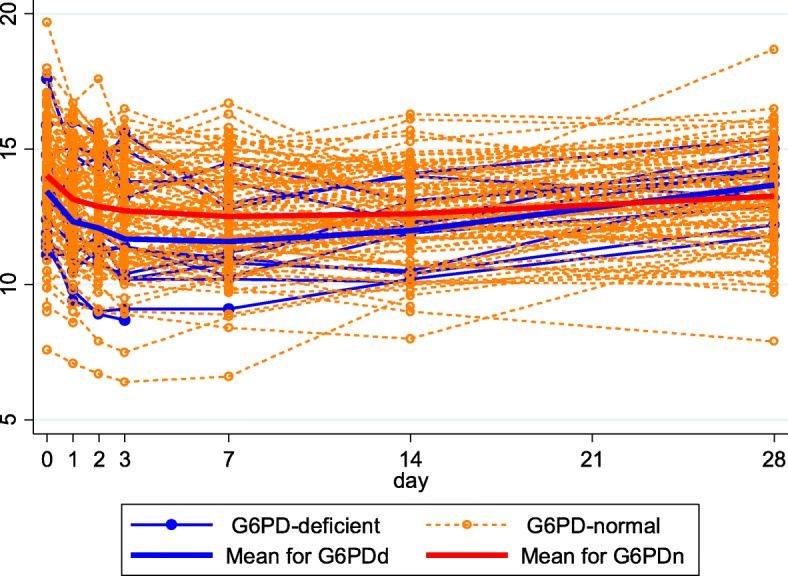
Haemoglobin dynamics of the HemoCue measured hemoglobin concentrations as a function of glucose-6-phosphate dehydrogenase (G6PD) status

For all patients, the mean HemoCue measured nadir Hb was 12.4 g/dL (D7) and rose to 13.1 g/dL by D28; Hb dynamics according to G6PD status are shown in Fig. 3. Being on DHAPP+SLDPQ, male, and of increasing age were associated with higher changes in Hb whereas reticulocyte dynamics were inversely associated with Hb dynamics (Table 2).

### Malaria attributable fall and Hb recovery

Post D7, the MAFt was higher in the G6PDd vs. the G6PDn group but the difference was not significantly different (*p* = 0.063): 1.81 vs. 0.57 g/dL, Δ = -1.25 (-0.75 ─ 2.56) g/dL. MAFt explanatory factors were D7 Hb concentration, treatment failure, both associated with lower a MAFt, and male sex (increased MAFt). The D28 Hb recovery rate was low, 35.7% (25/98), and more likely in males and older patients but less likely with higher D0 Hb concentrations (Table 2).

### Reticulocyte counts

**Fig. 4:**
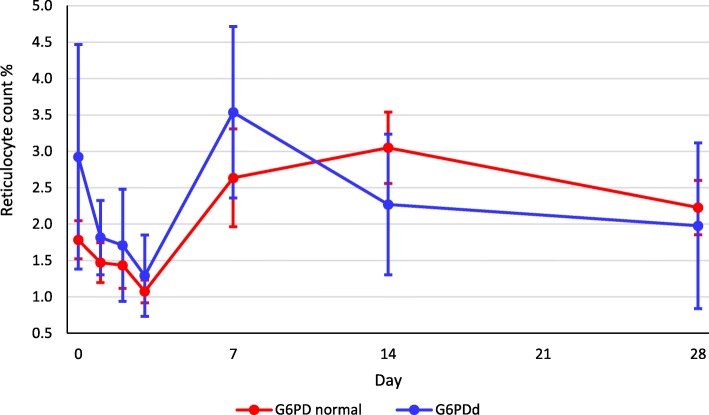
Reticulocyte count dynamics (mean and 95% confidence intervals) as a function of glucose-6-phosphate dehydrogenase status

The mean reticulocyte counts in the G6PDd and normal patients decreased initially on D1–3, increasing thereafter to peak on D7 for the G6PDd group and D14 for the G6PDn group (Fig. 4). The mean baseline reticulocyte count was significantly (*p* = 0.01) higher in G6PDd (2.9%) vs. G6PDn (1.8%) patients. There was an inverse relationship between the D0 Hb and reticulocyte dynamics (Table 2).

### Methaemoglobinemia

There was little change in the median metHb concentrations over time in both treatment arms; the highest value was 3.6% in a DHAPP-SLDPQ treated G6PDn patient (Additional file 2). Within the G6PDn group, metHb distributions were significantly different comparing D0 [1.3 (0.6–2)] vs: (i) D3 [1.6 (0.6–3.4)], *p* = 0.005), and (ii) D7 [1.6 (0.9–2.4)], *p* = 0.007.

### Clinical adverse events and reported symptoms over time

All patients tolerated their treatments well. Five patients developed late vomiting on D0, 1.2─2.4 h post drug administration that was probably drug related: four (DHAPP = 1) were aged 7 or 8 with grade 1 (mild) vomiting, and one was a DHAPP+SLDPQ treated, 35 years old male with grade 2 (moderate) vomiting. There were no AEs of clinical concern and no drug-related SAEs. Reported symptoms over time are shown in Additional file 3; no patient complained of itching, developing a rash, or passing dark urine.

Six (5.9%) of 102 patients had fractional falls in Hb ≥ 25% (25 ─ 33.1%, median 25.5%); five were G6PDn (one on DHAPP+SLDPQ) and one was G6PDd on DHAPP+SLDPQ.

## Discussion

We evaluated the WHO recommended dose of SLDPQ in DHAPP-treated Cambodian patients and have shown it was well tolerated and did not result in clinically significant haemolysis in moderately severe G6PDd Viangchan. Initial declines in Hb were not significantly different by G6PD status but the DHAPP+SLDPQ treated G6PDd patients had significantly lower mean D7 Hb by just over 1 g/dL.

The initial fall in Hb is the most critical period that determines whether a patient develops clinically severe anaemia to warrant a blood transfusion; in SE Asia, that risk in uncomplicated falciparum malaria is 1% [11]. The anxiety of malaria control programmes is that this risk would increase if SLDPQ were deployed. There is now growing evidence that although G6PDd patients have lower mean nadir Hb concentrations or greater Hb declines, these are not clinically threatening [16, 17, 22, 23].

Factors associated with a greater initial D7-D0 fall in our patients Hb were higher baseline Hb concentrations and parasitaemia, consistent with previous studies [10, 11, 30]. A higher parasite biomass results in greater malaria-related intra- and extravascular haemolysis and greater bone marrow suppression [31]. G6PDd was not a factor but this may have been due to small patient numbers. Interestingly, DHAPP+SLDPQ recipients had a lower D7-D0 decline, which is counter intuitive and difficult to explain. Tine et al. also found lower D7-D0 Hb declines in their adult African patients who received DHAPP+SLDPQ vs. DHAPP alone [18]. The D7 Hb difference (*p* = 0.01) between their G6PDd and G6PDn ACT+SLDPQ recipients was ~0.9 g/dL, a value similar to our study.

After D7, the mean rise in Hb (measured by the MAFt) was less in patients with LTF and those with a greater initial decline in Hb but higher in males vs. females. Hb recovery was also more likely in males and patients with higher baseline Hbs. Our high rate of LTF reconfirms the deleterious effect of multidrug resistant *P. falciparum* in the GMS [32–34] and this is probably increasing anaemia in malaria-affected communities. Our LTF rate was unaffected by SLDPQ; indeed, PQ at therapeutic doses does not possess blood stage activity against *P. falciparum* [35, 36].

Although G6PDd was not a factor determining MAFt or recovery, the G6PDd patients appear to have had a more rapid rate of Hb increase and an earlier time to mean peak reticulocytaemia. Bastiens et al detected a trend of a higher D7 reticulocytaemia in their G6PDd vs. G6PDn asymptomatic falciparum carriers treated with 0.25 or 0.4 mg/kg of PQ [16]. It is plausible that the G6PDd individuals mount a more robust bone marrow response following PQ but teasing out PQ vs. malaria related reticulocytosis might be challenging.

Important side effects of PQ are abdominal pain, vomiting and methaemoglobinemia; all are dose dependent and seen more often with doses ≥30 mg [37]. Very few patients reported abdominal pain and 5 had late vomiting. The maximum MetHb was 3.6%, 3 ─ 5 fold lower than the ~10–20% level that causes blue lips [38].

The main study limitation was the very small number (12) of G6PDd patients; thus, reducing substantially our statistical power. Nevertheless, we were able to show a significantly lower D7 Hb in DHAPP-SLDPQ treated G6PDd patients. We were only able to recruit three patients with Hbs ≤ 8 g/dL so more data are needed in moderate and severe anaemia because such patients are usually treated in the community. In Cambodia, ~5 and ~12% of malaria patients have Hbs < 5 and < 7 g/dL, respectively (analysis from [27]). Our follow up was short – 28 days – because our focus was safety, especially the first week during which the nadir Hb occurs. This also explains our low rate (~1/3) of Hb recovery, which may have improved with longer follow up [10, 11, 30]. As expected, most recruited patients were young male farmers, an important risk group in the GMS, so our data are applicable to other GMS countries with moderately severe G6PD variants and a similar malaria epidemiological characteristics as Cambodia [5, 39]. More work on SLDPQ is needed in other malaria endemic regions.

## Conclusions

Our study supports the use of SLDPQ in Cambodia and other GMS countries with similar G6PDd variants. Thanks partly to this study, Cambodia is now deploying SLDPQ and the design of an optimised, age-based SLDPQ regimen should facilitate its deployment down to the village level [40]. SLDPQ deployment should be carried out in conjunction with pharmacovigilance [40].

## Additional files

Additional file 1: List of G6PD status with G6PD enzyme activity (measured in a subset), baseline reticulocyte count and haemoglobin concentration and the result of the fluorescent spot test. (XLSX 13 kb)

Additional file 2: Methaemoglobin concentration over time, expressed as the % of total haemoglobin. Within the G6PDn group, metHb distributions were significantly different for: (i) D3 [1.6 (0.6-3.4)] vs. D0 [1.3 (0.6-2)] *p*=0.005), and (ii) D7 [1.6 (0.9-2.4)] vs. D0 (*p*=0.007). (DOCX 13 kb)

Additional file 3: Symptoms reported and selected signs detected during follow up and selected signs suggestive of anaemia. (DOCX 30 kb)

## Abbreviations

ACTs: artemisinin-based combination therapies
AE: adverse event
AHA: acute haemolytic anaemia
ALT: alanine aminotransferase
AST: aspartate aminotransferase
CRFs: case record forms
D: Day
DHAPP: dihydroartemisinin piperaquine
G6PDd: glucose-6-phosphate dehydrogenase deficiency
GMS: Greater Mekong Sub Region
Hb: haemoglobin
LTF: late treatment failure
MAFt: total malaria attributable fall
metHb: methaemoglobin
NE: northeast
*Pf*: *Plasmodium falciparum*
PQ: primaquine
*PV*: *Plasmodium vivax*
SAE: serious adverse event
SD: standard deviation
SE: southeast
SLDPQ: single low dose primaquine
WHO: World Health Organisation
