# Supplementary material for: The tolerability of single low dose primaquine in glucose-6-phosphate deficient and normal falciparum-infected Cambodians
Source: BMC Infect Dis. 2019 Mar 12;19:250. doi: 10.1186/s12879-019-3862-1 (PMC6419451; doi:10.1186/s12879-019-3862-1)
Supplement: Supplementary file 2 — Methaemoglobin concentration over time, expressed as the % of total haemoglobin. Within the G6PDn group, metHb distributions were significantly different for: (i) D3 [1.6 (0.6-3.4)] vs. D0 [1.3 (0.6-2)] p=0.005), and (ii) D7 [1.6 (0.9-2.4)] vs. D0 (p=0.007). (DOCX 13 kb) [file 12879_2019_3862_MOESM2_ESM.docx]

Additional file 2. Methaemoglobin concentration over time, expressed as the % of total haemoglobin. Within the G6PDn group, metHb distributions were significantly different for: (i) D3 [1.6 (0.6-3.4)] vs. D0 [1.3 (0.6-2)] p=0.005), and (ii) D7 [1.6 (0.9-2.4)] vs. D0 (p=0.007).

|  | **Minimum** | **Lower quartile** | **Median** | **Upper quartile** | **Maximum** |
| --- | --- | --- | --- | --- | --- |
| ***DHAPP* *alone*** |  |  |  |  |  |
| Day 0 | 0.7 | 1.3 | 1.5 | 1.8 | 1.9 |
| Day 1 | 0.8 | 1.35 | 1.5 | 1.65 | 1.9 |
| Day 2 | 0.8 | 1.3 | 1.5 | 1.6 | 1.9 |
| Day 3 | 0.6 | 1.3 | 1.5 | 1.7 | 1.9 |
| Day 7 | 0.8 | 1.4 | 1.6 | 1.8 | 1.9 |
| Day 14 | 0.9 | 1.4 | 1.6 | 1.8 | 1.9 |
| Day 28 | 0.9 | 1.4 | 1.7 | 1.8 | 2 |
| ***DHAPP*+*SLDPQ*** |  |  |  |  |  |
| Day 0 | 0.6 | 1.2 | 1.3 | 1.7 | 2 |
| Day 1 | 0.8 | 1.2 | 1.5 | 1.8 | 3.6 |
| Day 2 | 0.8 | 1.3 | 1.5 | 1.7 | 3.4 |
| Day 3 | 0.6 | 1.4 | 1.6 | 1.8 | 3.4 |
| Day 7 | 0.9 | 1.4 | 1.6 | 1.8 | 2.4 |
| Day 14 | 0.9 | 1.3 | 1.5 | 1.8 | 2.1 |
| Day 28 | 0.9 | 1.3 | 1.6 | 1.8 | 2 |

DHAPP+SLDPQ – dihydroartemisinin piperaquine & single low dose primaquine
