# Supplementary material for: The tolerability of single low dose primaquine in glucose-6-phosphate deficient and normal falciparum-infected Cambodians
Source: BMC Infect Dis. 2019 Mar 12;19:250. doi: 10.1186/s12879-019-3862-1 (PMC6419451; doi:10.1186/s12879-019-3862-1)
Supplement: Supplementary file 3 — Symptoms reported and selected signs detected during follow up and selected signs suggestive of anaemia. (DOCX 30 kb) [file 12879_2019_3862_MOESM3_ESM.docx]

Additional file 3. Symptoms reported and selected signs detected during follow up and selected signs suggestive of anaemia.

|  | **D1** | | | **D2** | | | **D3** | | | **D7** | | | **D14** | | | **D28** | | |
| --- | --- | --- | --- | --- | --- | --- | --- | --- | --- | --- | --- | --- | --- | --- | --- | --- | --- | --- |
|  | **DHAPP (n=52)** | **DHAPP+PQ (n=55)** | **DHAPP (n=51)** | | **DHAPP+PQ (n=54)** | **DHAPP (n=50)** | | **DHAPP+PQ (n=54)** | **DHAPP (n=50)** | | **DHAPP+PQ (n=55)** | **DHAPP (n=48)** | | **DHAPP+PQ (n=50)** | **DHAPP (n=38)** | | **DHAPP+PQ (n=47)** |  |
| General wellbeing* | 3 (2-4) | 3 (2-4) | 3 (2-4) | | 3 (3-5) | 4 (3-5) | | 4 (3-5) | 4 (3-5) | | 4 (3-5) | 4 (2-5) | | 4 (4-5) | 5 (2-5) | | 4 (2-5) |  |
| Fever | 0 (0)† | 4 (7.3) | 0 (0) | | 1 (1.9) | 0 (0) | | 1 (1.9) | 0 (0) | | 0 (0) | 1 (2.1) | | 1 (2.0) | 3 (7.9) | | 3 (6.4) |  |
| Chills | 1 (0) | 5 (9.1) | 0 (0) | | 0 (0) | 0 (0) | | 0 (0) | 0 (0) | | 0 (0) | 1 (2.1) | | 1 (2.0) | 3 (7.9) | | 3 (6.4) |  |
| Headache | 4 (7.7) | 15 (27.3) | 2 (3.9) | | 7 (13.0) | 1 (2.0) | | 3 (5.6) | 1 (2.0) | | 1 (1.8) | 1 (2.1) | | 3 (6.0) | 4 (10.5) | | 4 (8.5) |  |
| Anorexia | 0 (0) | 0 (0) | 0 (0) | | 0 (0) | 0 (0) | | 0 (0) | 0 (0) | | 0 (0) | 0 (0) | | 0 (0) | 2 (5.3) | | 0 (0) |  |
| Nausea | 1 (1.9) | 0 (0) | 0 (0) | | 0 (0) | 0 (0) | | 0 (0) | 0 (0) | | 0 (0) | 0 (0) | | 0 (0) | 0 (0) | | 0 (0) |  |
| Vomiting | 0 (0) | 1 (1.8) | 0 (0) | | 1 (1.9) | 0 (0) | | 0 (0) | 0 (0) | | 0 (0) | 0 (0) | | 0 (0) | 0 (0) | | 0 (0) |  |
| Abdominal pain | 1 (1.9) | 3 (5.5) | 1 (2.0) | | 0 (0) | 1 (2.0) | | 1 (1.9) | 0 (0) | | 0 (0) | 0 (0) | | 0 (0) | 0 (0) | | 0 (0) |  |
| Diarrhoea episodes | 0 (0) | 1 (1.8) | 0 (0) | | 1 (1.9) | 0 (0) | | 0 (0) | 0 (0) | | 0 (0) | 0 (0) | | 1 (2.0) | 0 (0) | | 0 (0) |  |
| Palpitations | 0 (0) | 4 (7.3) | 0 (0) | | 2 (3.7) | 0 (0) | | 1 (1.9) | 0 (0) | | 0 (0) | 0 (0) | | 1 (2.0) | 1 (2.1) | | 1 (2.6) |  |
| Cough | 3 (5.8) | 3 (5.5) | 0 (0) | | 3 (5.6) | 0 (0) | | 3 (5.6) | 0 (0) | | 1 (1.8) | 0 (0) | | 3 (6.0) | 1 (2.1) | | 2 (4.3) |  |
| SOBOE† | 1 (1.9) | 0 (0) | 0 (0) | | 0 (0) | 0 (0) | | 1 (1.9) | 0 (0) | | 0 (0) | 0 (0) | | 0 (0) | 0 (0) | | 0 (0) |  |
| Muscle aches | 0 (0) | 4 (7.3) | 0 (0) | | 0 (0) | 0 (0) | | 0 (0) | 1 (2.0) | | 0 (0) | 0 (0) | | 0 (0) | 1 (2.1) | | 4 (8.5) |  |
| Passing normal colour urine | 52 (100) | 55 (100) | 51 (100) | | 54 (100) | 50 (100) | | 54 (100) | 50 (100) | | 55 (100) | 48 (100) | | 50 (100) | 38 (100) | | 47 (100) |  |
| Skin rash | 0 (0) | 0 (0) | 0 (0) | | 0 (0) | 0 (0) | | 0 (0) | 0 (0) | | 0 (0) | 0 (0) | | 0 (0) | 0 (0) | | 0 (0) |  |
| Itching | 0 (0) | 0 (0) | 0 (0) | | 0 (0) | 0 (0) | | 0 (0) | 0 (0) | | 0 (0) | 0 (0) | | 0 (0) | 0 (0) | | 0 (0) |  |
| Pale conjunctivae | 0 (0) | 0 (0) | 0 (0) | | 0 (0) | 0 (0) | | 0 (0) | 0 (0) | | 0 (0) | 0 (0) | | 0 (0) | 0 (0) | | 0 (0) |  |
| Pale palms | 0 (0) | 0 (0) | 0 (0) | | 0 (0) | 0 (0) | | 0 (0) | 0 (0) | | 0 (0) | 0 (0) | | 0 (0) | 0 (0) | | 0 (0) |  |
| Pale tongue | 0 (0) | 0 (0) | 0 (0) | | 0 (0) | 0 (0) | | 0 (0) | 0 (0) | | 0 (0) | 0 (0) | | 0 (0) | 0 (0) | | 0 (0) |  |
| Hillmen score¶ | 3 (1-3) | 2 (1-3) | 2 (1-3) | | 2 (1-3) | 2 (1-3) | | 2 (1-3) | 2 (1-2) | | 2 (1-3) | 2 (1-3) | | 2 (1-3) | 1 (1-3) | | 1 (1-2) |  |

* on a scale of 1-5, data are median (range)

† N (%) of patients reporting given symptom

‡ shortness of breath on exertion

¶ on a scale of 1─12, data are median, full range
